# Correction: Self-quenched ferrocenyl diketopyrrolopyrrole organic nanoparticles with amplifying photothermal effect for cancer therapy

**DOI:** 10.1039/d3sc90166a

**Published:** 2023-09-07

**Authors:** Pingping Liang, Qianyun Tang, Yu Cai, Gongyuan Liu, Weili Si, Jinjun Shao, Wei Huang, Qi Zhang, Xiaochen Dong

**Affiliations:** a Key Laboratory of Flexible Electronics (KLOFE), Institute of Advanced Materials (IAM), Jiangsu National Synergetic Innovation Center for Advanced Materials (SICAM), Nanjing Tech University (Nanjing Tech) 30 South Puzhu Road Nanjing 211816 China iamxcdong@njtech.edu.cn iamwhuang@njtech.edu.cn; b School of Pharmaceutical Sciences, Nanjing Tech University (Nanjing Tech) Nanjing China zhangqi@njtech.edu.cn

## Abstract

Correction for ‘Self-quenched ferrocenyl diketopyrrolopyrrole organic nanoparticles with amplifying photothermal effect for cancer therapy’ by Pingping Liang *et al.*, *Chem. Sci.*, 2017, **8**, 7457–7463, https://doi.org/10.1039/C7SC03351F.

It has come to our attention that some errors have been found in Fig. S11. The H&E staining images of the Heart, Liver, and Lung (Saline group) and Lung (Saline + Laser group) in Fig. S11 of the published work were misused when editing the photos. The mistake was found by the authors after the paper was published online. The correct Fig. S11 is given below. The results and conclusions of this paper are not affected by this correction.


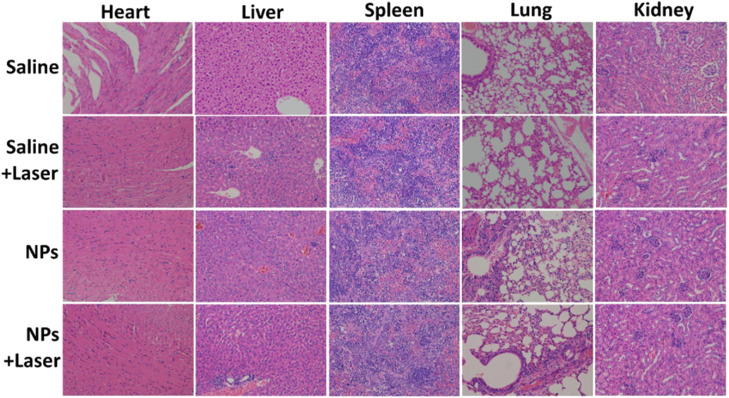
 **Fig. S11** Photographs of H&E stained major organs including heart, liver, spleen, lung and kidney obtained from four groups after 18 days of treatment.

 

The Royal Society of Chemistry apologises for these errors and any consequent inconvenience to authors and readers.

## Supplementary Material

